# Cross-Modality Person Re-Identification Method with Joint-Modality Generation and Feature Enhancement

**DOI:** 10.3390/e26080681

**Published:** 2024-08-13

**Authors:** Yihan Bi, Rong Wang, Qianli Zhou, Zhaolong Zeng, Ronghui Lin, Mingjie Wang

**Affiliations:** 1 School of Information and Cyber Security, People’s Public Security University of China, Beijing 100038, China; rcbyh2023@126.com (Y.B.);; 2 Key Laboratory of Security Prevention Technology and Risk Assessment of Ministry of Public Security, Beijing 100038, China; 3 Beijing Public Security Bureau, Beijing 100038, China

**Keywords:** person re-identification, visible–infrared image, modality generation, feature enhancement, gradient centralization

## Abstract

In order to minimize the disparity between visible and infrared modalities and enhance pedestrian feature representation, a cross-modality person re-identification method is proposed, which integrates modality generation and feature enhancement. Specifically, a lightweight network is used for dimension reduction and augmentation of visible images, and intermediate modalities are generated to bridge the gap between visible images and infrared images. The Convolutional Block Attention Module is embedded into the ResNet50 backbone network to selectively emphasize key features sequentially from both channel and spatial dimensions. Additionally, the Gradient Centralization algorithm is introduced into the Stochastic Gradient Descent optimizer to accelerate convergence speed and improve generalization capability of the network model. Experimental results on SYSU-MM01 and RegDB datasets demonstrate that our improved network model achieves significant performance gains, with an increase in Rank-1 accuracy of 7.12% and 6.34%, as well as an improvement in mAP of 4.00% and 6.05%, respectively.

## 1. Introduction

Person re-identification is an important issue in the field of computer vision [1], aimed at retrieving specific pedestrians from multiple non-overlapping cameras [2]. Traditional research on person re-identification mainly focuses on the application of technology under single modality, and most of the research is limited to the scene with sufficient light. With the continuous improvement in video surveillance security requirements, cameras with infrared modality switching function are gradually becoming popular and are applied in order to effectively cope with the limited working effect of visible cameras in night-time or harsh weather conditions. Aiming at the problem of different day and night lighting conditions, visible–infrared cross-modality person re-identification has been proposed [3].

Differently from traditional person re-identification under single modality, visible-infrared cross-modality person re-identification mainly studies the retrieval and matching of visible and infrared images of specific pedestrians in different horizons [4]. Due to the fact that visible images have three channels containing visible-light color information: red (R), green (G), and blue (B), while infrared images only have one channel containing near-infrared light intensity information, there is a fundamental difference in the images captured under the two modalities [5].

To alleviate this difference, the studies have been carried out based on three types of methods, including representation learning [6,7,8], metric learning [9,10,11], and modality conversion [12,13,14]. However, these methods tend to conduct cross-modality learning from the direct mapping of the two original modalities of visible images and infrared images, which makes the network model sensitive to parameters, difficult to converge, and requires a large amount of computational resources. Furthermore, existing methods fail to fully extract key channel features and spatial features of pedestrians in visible images and infrared images. These issues all result in low recognition accuracy of cross-modality person re-identification methods.

To address these issues, we propose a novel cross-modality person re-identification method based on the Attention Generalized mean pooling with Weighted triplet loss [15] (AGW) network model, which integrates modality generation and feature enhancement. The proposed method fully extracts shared features between visible images and infrared images, extracts more representative and generalized pedestrian features, accelerates the convergence speed of the network model, and improves the accuracy of cross-modality person re-identification. Our main contributions can be summarized as follows:

- A lightweight network is used to generate intermediate modalities between visible images and infrared images as auxiliary modalities, enabling the network model to fully extract shared features between visible images and infrared images;
- The feature enhancement method is utilized for optimizing feature extraction, and key feature information in pedestrian images is weighted and enhanced sequentially from both channel and spatial dimensions to enhance the efficiency and representation ability of the network model in utilizing pedestrian features;
- The optimization strategy of centralizing gradient vectors is introduced to improve the generalization ability and training efficiency of the network model;
- The experimental results on SYSU-MM01 and RegDB datasets show the superiority of the proposed method.

## 2. Related Work

The main challenge faced by cross-modality person re-identification is the modality differences between visible images and infrared images [16]. From the perspective of reducing the modality differences between visible images and infrared images, the visible-infrared cross-modality person re-identification methods can be summarized into three types: methods based on representation learning, methods based on metric learning, and methods based on modality conversion [3].

Methods based on representation learning mainly study how to design a reasonable network model architecture to extract discriminative and robust pedestrian features shared by visible images and infrared images, in order to reduce the modality differences between visible images and infrared images. In 2017, Wu et al. [6] proposed a deep-zero-padding data preprocessing method to train a single-stream network model. They first proposed cross-modality person re-identification and constructed the publicly available cross-modality person re-identification dataset SYSU-MM01; Yu et al. [7] proposed a dual-stream multi-branch network model based on fully correlated attention, which improves the color style robustness of the network model through a color-randomization data-augmentation algorithm, and enhances the discriminability of the extracted features by extracting multi-scale global features and local features; Fan et al. [8] proposed a clustering learning network model based on feature enhancement, which mines and enhances the subtle features of visible images and infrared images through global features and local features, and combines multi-level joint-clustering learning strategies to minimize modality differences and intra-class changes.

Methods based on metric learning mainly study how to reduce the modality differences between visible images and infrared images by designing a reasonable loss function to measure the similarity between two pedestrian images. Wang et al. [9] proposed a dual-path attention network model that enhances feature extraction through spatial dependencies between local features in pedestrian feature maps, and achieves center and boundary constraints on each class distribution through the proposed cross-modality dual-constraint loss; Zou et al. [10] proposed a heterogeneous center triplet loss based on angular distance, which not only solves the problem of selecting abnormal samples in traditional triplet loss, but also reduces the computational complexity of the network model; Zhang et al. [11] designed modality-consistency constraint loss and feature-center constraint loss, so that the modality-consistency constraint loss guides the network model to learn the invariant features between modalities, and the feature-center constraint loss supervises the network model to reduce intra-class feature differences and increase inter-class feature differences.

Methods based on modality conversion mainly study how to realize the mutual conversion of visible images and infrared images, utilizing a generative adversarial network [17] (GAN), and transform the cross-modality person re-identification into person re-identification under single modality to reduce the modality differences between visible images and infrared images. Wang et al. [12] proposed a double-layer difference-reduction learning method that decomposes mixed modalities and appearance differences. The difference-reduction subnetworks of image level and feature level are cascaded and jointly optimized in an end-to-end manner, while constraining modality and appearance differences; Wang et al. [13] proposed an end-to-end aligned generative adversarial network model, which reduces modality differences through a pixel alignment module and feature alignment module, and ensures identity consistency through a joint discrimination module; Zhang et al. [14] proposed a teacher–student generative adversarial network model based on different domains. By generating visible images into infrared images, the pre-trained teacher model generates feature maps to guide the student model in extracting discriminative features in the backbone network.

The aforementioned methods [6,7,8,9,10,11,12,13,14] address the issue of matching visible images and infrared images, to a certain extent. However, some challenges still remain unsolved, like low pedestrian-feature utilization efficiency and poor representation ability, insufficient network-model generalization ability, and slow convergence speed in cross-modality person re-identification. It is essential for the task to extract shared features of two modalities to effectively reduce the modality differences, and extract more representative pedestrian key channel features and spatial features and find a novel way to improve the training efficiency. Therefore, we propose a novel cross-modality person re-identification method to address the challenges above.

## 3. Methods

In this section, we first outline the overall framework of the proposed method in Section 3.1. Secondly, in Section 3.2, we explain the working principle of the lightweight modality generator [18] used in the proposed method. Then, in Section 3.3, we introduce the Convolutional Block Attention Module [19] (CBAM) incorporated into the feature extraction part of this work. Next, in Section 3.4, we describe the Gradient Centralization [20] (GC) optimization strategy adopted in the proposed method. Finally, in Section 3.5, we present all the loss functions that make up the network model.

### 3.1. Framework of the Proposed Method

**Figure 1:**
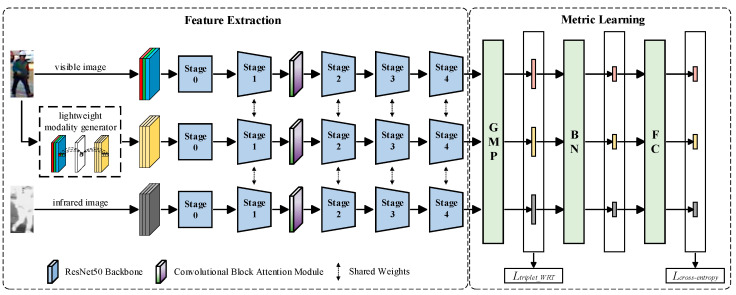
Overall architecture of the proposed method. Among them, the initial Stage 0 includes the initial convolutional layer, batch normalization (BN) layer, ReLU layer, and Max pooling layer.

On the basis of the AGW [15] network model, the proposed method consists of two parts: feature extraction and metric learning. As shown in Figure 1, the proposed method adopts a three-branch structure with ResNet50 [21], which embeds non-local neural networks [22] in Stage 2 and Stage 3, as the backbone network.

In the part of feature extraction, we input visible images into the lightweight modality generator for dimensionality reduction and augmentation, generating intermediate modalities between visible images and infrared images. Then, we jointly input them into the backbone network that shares weight parameters with other parts, except for the independent parameterized Stage 0, for feature extraction. In particular, we embedded the Convolutional Block Attention Module into Stage 1, weighting and enhancing key feature information sequentially from both channel and spatial dimensions. In the part of metric learning, all feature maps are constrained by the Generalized Mean pooling [23] (GeM) layer to output feature vectors and calculate the Weighted Regularization Triplet loss [15] (WRT). Afterwards, we used batch normalization [24] (BN) to normalize the feature vectors and mapped them using fully connected (FC) layers to calculate cross-entropy loss.

The network structure and overall process of the proposed method are shown in Figure 1, where GMP represents Generalized Mean pooling, BN represents batch normalization, and FC represents the fully connected layer. $L_{\mathrm{triplet}\text{\_}\mathrm{WRT}}$ and $L_{\mathrm{cross}-\mathrm{entropy}}$ represent the Weighted Regularization Triplet loss and cross-entropy loss, respectively.

### 3.2. Lightweight Modality Generator

The intermediate modality between visible images and infrared images can coordinate the connection between visible images and infrared images and establish a link, enabling the network model to fully extract the shared features between visible images and infrared images and thereby effectively reduce the modality differences between visible images and infrared images. The lightweight modality generator we utilized comprises two convolutional layers with kernel size 1×1 and a ReLU [25] activation layer. Compared to methods that use other auxiliary structures, such as generative adversarial network, the lightweight modality generator is more efficient and easy to optimize.

Using visible images as input, the intermediate modalities can be obtained from Equation (1). Among them, *V* represents the visible images, *M* represents the intermediate modalities, and *g* represents the lightweight modality generator, respectively.

(1) $$M=g\left(V\right)$$

**Figure 2:**
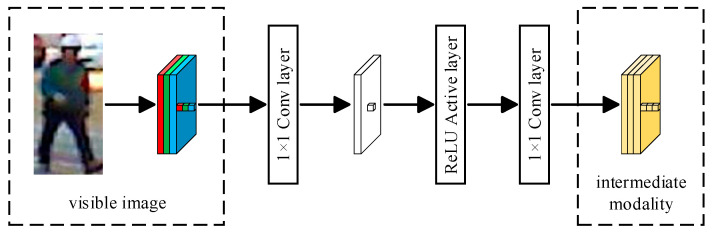
Structure of the lightweight modality generator.

Specifically, the steps for generating intermediate modalities using the lightweight modality generator are shown in Figure 2. Firstly, the original three-channel visible image is mapped to a single-channel image after passing through the first 1×1 convolutional layer. This procedure compresses the features within the visible image into one channel. Subsequently, the single-channel image is passed into the ReLU activation layer, which effectively enhances the nonlinear expression capability. Finally, the single-channel image that passes through the ReLU activation layer is mapped to the three-channel intermediate modality image through another 1×1 convolutional layer. This procedure restores the number of channels in the image and preserves the crucial features extracted through the first two steps.

By generating intermediate modalities between visible images and infrared images, we effectively extracted shared features between these two images, thereby reducing the modality differences between visible images and infrared images. Using the intermediate modalities as auxiliary modalities, we connected visible images and infrared images, promoting the sharing of their features and making cross-modality learning easier.

### 3.3. Convolutional Block Attention Module

**Figure 3:**
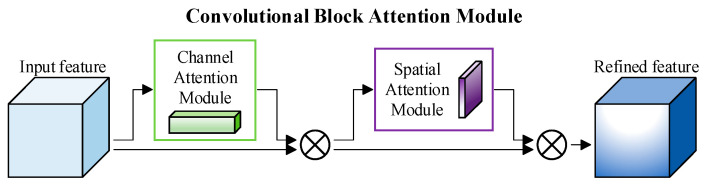
Overview of Convolutional Block Attention Module.

The introduction of attention mechanism can enable network models to focus on key information and features in data, thereby improving the efficiency and representation ability of network models in utilizing information. As a lightweight attention module, the Convolutional Block Attention Module can improve the feature representation ability and performance of network models in situations where training data and computing resources are limited. As shown in Figure 3, the Convolutional Block Attention Module consists of two modules: the channel attention module and the spatial attention module. Attention weights are inferred along the channel and spatial dimensions, and then multiplied with the input feature map to perform feature refinement operations.

Specifically, for the input feature map *F*, we pass it through the channel attention module to obtain the channel attention map $M_{C}(F)$, which represents the evaluation of the importance of each channel in the input feature map *F* by the channel attention module. The operational steps of the channel attention module are shown in Equation (2), where σ represents the sigmoid function, *AvgPool* represents the global average-pooling operation, and *MaxPool* represents the global maximum-pooling operation.

(2) $$M_{C}\left(F\right)=\sigma \left(MLP\left(AvgPool\left(F\right)\right)\right)+MLP\left(MaxPool\left(F\right)\right)$$

We multiply the channel attention map $M_{C}(F)$ with the input feature map *F* of the channel attention module to obtain the input feature map *F*′ of the spatial attention module, as shown in Equation (3).

(3) $$F^{\prime }=M_{C}\left(F\right)⊗F$$

After passing it through the spatial attention module, we obtain the spatial attention map $M_{S}\left(F^{\prime }\right)$, which represents the evaluation of the importance of each pixel in the input feature map *F*′ by the spatial attention module. The operational steps of the spatial attention module are shown in Equation (4). Among them, σ represents the sigmoid function, $f^{\left(7\times 7\right)}$ represents the convolution operation with a filter size of 7×7, *AvgPool* represents the global average-pooling operation, and *MaxPool* represents the global maximum-pooling operation.

(4) $$M_{S}\left(F^{\prime }\right)=\sigma \left(f^{\left(7\times 7\right)}\left(\left[AvgPool\left(F^{\prime }\right);MaxPool\left(F^{\prime }\right)\right]\right)\right)$$

We multiply the spatial attention map $M_{S}(F’)$ with the input feature map *F*′ of the spatial attention module to obtain the output feature map *F*″ of the input feature map *F* after being processed by the Convolutional Block Attention Module, as shown in Equation (5).

(5) $$F^{\prime \prime }=M_{S}\left(F^{\prime }\right)⊗F^{\prime }$$

**Figure 4:**
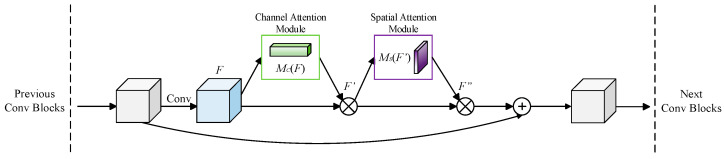
Residual structure of CBAM-ResNet.

We embed the Convolutional Block Attention Module after the third residual block in Stage 1 of the backbone network ResNet50, so that the improved network model can weight and enhance key feature information sequentially from both channel and spatial dimensions in sequence, thereby extracting more representative and generalized pedestrian features. The residual structure after adding the Convolutional Block Attention Module (CBAM) is shown in Figure 4.

### 3.4. Gradient Centralization

**Figure 5:**
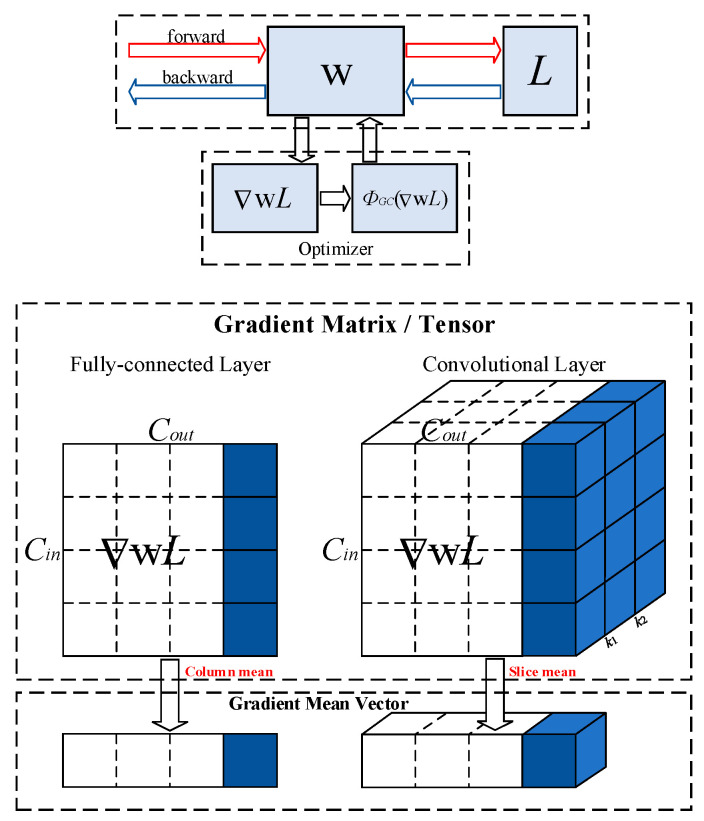
Diagram of Gradient Centralization.

As an effective gradient optimization strategy, Gradient Centralization directly acts on the weight gradient, constraining the loss function in the network model by calculating the mean of the gradient vector, accelerating the convergence speed of the network model, improving the generalization ability of the network model, and enhancing the disturbance resistance of the loss function, as shown in Figure 5. Therefore, introducing Gradient Centralization into the Stochastic Gradient Descent [26] (SGD) optimizer regularizes the weight space and output-feature space, making the training process of the network model more stable and efficient.

Taking the fully connected layer as an example, in the case of obtaining the backpropagation gradient of the network model, the average value of each column vector in the gradient matrix is calculated, and then the average value is removed from each column vector to achieve centralization of each column vector. Finally, the centralized gradient matrix is returned, as shown in Equation (6):

(6) $$\Phi_{GC}\left(\nabla w_{p}L\right)=\nabla w_{p}L-\frac{1}{n}\sum_{q=1}^{n}\nabla w_{p,q}L$$

Among them, $\nabla w_{p}L$ represent gradients, and $w_{p}$ represent weight vectors; p represents the *p*-th column vector in the gradient matrix, and q represents the *q*-th element in the *p*-th column vector.

### 3.5. Loss Function

We use the Weighted Regularization Triplet loss and cross-entropy loss as the metric loss and ID loss of the network model, respectively. The total loss function of the network model is shown in Equation (7), where $L_{\mathrm{triplet}\text{\_}\mathrm{WRT}}$ represents the Weighted Regularization Triplet loss and $L_{\mathrm{cross}-\mathrm{entropy}}$ represents the cross-entropy loss.

(7) $$L_{\mathrm{total}}=L_{triplet\_WRT}+L_{\mathrm{cross}-\mathrm{entropy}}$$

The Weighted Regularization Triplet loss can maintain the optimization of the relative distance between positive and negative sample pairs in traditional difficult samples for sampling triplet loss [27] without introducing any additional parameters, and has strong flexibility and adaptability. The calculation process of the Weighted Regularization Triplet loss is shown in Equation (8). Among them, (i,j,k) represents the difficult samples of sampling triplets within each training batch, *P* represents the positive sample set, *N* represents the negative sample set, $d_{\mathrm{ij}}^{p}$ represents the distance between the least similar positive sample pairs, $d_{\mathrm{ik}}^{n}$ represents the distance between the most similar negative sample pairs, $d^{p}$ represents the distance between each positive sample pair, and $d^{n}$ represents the distance between each negative sample pair.

(8) $$\begin{array}{c} L_{triplet\_WRT}\left(i,j,k\right)=\log\left(1+\exp\left(w_{i}^{p}d_{ij}^{p}-w_{i}^{n}d_{ik}^{n}\right)\right)\\ w_{i}^{p}=\frac{\exp\left(d_{ij}^{p}\right)}{\sum_{p\in P}\exp\left(d^{p}\right)}\\ w_{i}^{n}=\frac{\exp\left(-d_{ik}^{n}\right)}{\sum_{n\in N}\exp\left(-d^{n}\right)} \end{array}$$

Cross-entropy loss anomaly is used in image classification tasks to evaluate the predictive performance of a network model by measuring the degree of similarity between its predictions and real data. The calculation process of cross-entropy loss is shown in Equation (9). Among them, *N* represents the number of samples, *K* represents the number of categories, *y_i_*_,*k*_ represents the true category of the *i*-th sample, as *k*, and *p_i_*_,*k*_ represents the probability of the predicted category of the *i*-th sample, as *k*.

(9) $$L_{\mathrm{cross}-entropy}=-\frac{1}{N}\sum_{i=1}^{N}\sum_{k=1}^{K}y_{i,k}\ln p_{i,k}$$

We achieve constraints on the network model by using a joint supervision approach of the Weighted Regularization Triplet loss and cross-entropy loss.

## 4. Experiment and Analysis

### 4.1. Datasets and Evaluation Metric

We conducted experiments on two cross-modality person re-identification datasets, SYSU-MM01 [6] and RegDB [28], as shown in Table 1. Among them, the SYSU-MM01 dataset was captured by Sun Yat-sen University using 6 cameras, including 30,071 visible images and 15,792 infrared images of 491 pedestrians. The training set includes 11,909 infrared images and 22,258 visible images from 395 different pedestrians. In the test set, 3803 infrared images of 96 pedestrians were used as the query, and 301 randomly selected visible images were used as the gallery. The RegDB dataset was captured by a dual camera system consisting of a visible camera and a thermal imaging camera, containing 412 different pedestrians, each with 10 visible images and 10 infrared images. The training set includes 206 pedestrians, 2060 visible images, and 2060 infrared images. The test set includes 206 pedestrians, 2060 visible images, and 2060 infrared images.

We use the commonly used Cumulative Match Characteristic (CMC) and mean Average Precision [29] (mAP) in retrieval tasks as evaluation metrics for the network model. Among them, CMC is used to measure the network model’s ability to correctly identify target pedestrians during the retrieval process. It can intuitively reflect the performance of the network model by calculating the correct matching rate under different rankings. mAP is used to measure the average accuracy of network models in retrieving correct pedestrian identities. It can comprehensively evaluate the performance of the network model in all query scenarios by calculating the average accuracy of each retrieval in all queries.

### 4.2. Experimental Settings

The experiment was implemented on NVIDIA TITAN V GPU (manufacturer NVIDIA, sourced from Beijing, China) using PyTorch deep learning framework version 1.4.0. We used ResNet50, which removes the average pooling layer and fully connected layer, as the backbone network, and set the convolution step size of the last layer to 1. During the training process, we enhanced the image data through random cropping and horizontal flipping operations, and adjusted the image size to 288×144. The number of epochs was set to 80, and the batch size was set to 72, including 24 visible images, 24 infrared images, and 24 intermediate modality images of 6 pedestrians. As shown in Equation (10), we used the dynamic learning rate and the Stochastic Gradient Descent optimizer with Gradient Centralization to update the gradient of the network model. The weight decay of the optimizer is set to $5\times 10^{-4}$, and the momentum factor is set to 0.9.

(10) $$lr\left(\mathrm{epoch}\right)=\left\{\begin{array}{cc} 0.1\times \frac{epoch+1}{10}, & epoch<10\\ 0.1, & 10\leq epoch<20\\ 0.01, & 20\leq epoch<50\\ 0.001, & epoch\geq 50 \end{array}\right.$$

### 4.3. Comparison with Existing Methods

We compared the performance of our proposed method with existing cross-modality person re-identification methods on the SYSU-MM01 and RegDB datasets, and the results are shown in Table 2. It can be seen that, compared with the baseline method, the proposed method has improved recognition accuracy on both the SYSU-MM01 and RegDB datasets, proving the effectiveness of the improved method. Compared with the existing cross-modality person re-identification methods in the table, the proposed method has significantly improved recognition accuracy on the SYSU-MM01 and RegDB datasets, proving its effectiveness.

### 4.4. Ablation Study

#### 4.4.1. Using Lightweight Modality Generator

Visible images were input into the lightweight modality generator for dimensionality reduction and augmentation, generating intermediate modalities between visible images and infrared images, and were then input into the network model, together with visible images and infrared images. The performance evaluation results of the network model are shown in Table 3. It can be seen that the network model using the lightweight modality generator shows significant performance improvements on both the SYSU-MM01 and RegDB datasets. Among them, on the SYSU-MM01 dataset, Rank-1 improved by 5.47% and mAP improved by 1.16%; on the RegDB dataset, Rank-1 improved by 4.09% and mAP improved by 2.39%.

#### 4.4.2. Incorporating Convolutional Block Attention Module

The Convolutional Block Attention Module was embedded into the backbone network ResNet50, and the performance evaluation results of the network model are shown in Table 4. It can be seen that the network model embedded with the Convolutional Block Attention Module shows modest performance improvements on both the SYSU-MM01 and RegDB datasets. Among them, on the SYSU-MM01 dataset, Rank-1 improved by 3.89% and mAP improved by 0.82%; on the RegDB dataset, Rank-1 improved by 3.01% and mAP improved by 2.10%.

#### 4.4.3. Introducing Gradient Centralization

The Gradient Centralization algorithm was introduced into the Stochastic Gradient Descent optimizer, and the performance evaluation results of the network model are shown in Table 5. It can be seen that the network model introducing Gradient Centralization shows a slight improvement in performance on both the SYSU-MM01 and RegDB datasets. Among them, on the SYSU-MM01 dataset, Rank-1 improved by 0.09% and mAP improved by 0.23%; on the RegDB dataset, Rank-1 improved by 0.31% and mAP improved by 0.37%.

#### 4.4.4. Ablation Experiments to Verify the Effectiveness of 3 Modules

To verify the effectiveness of various parts of the network model, we conducted ablation experiments on the SYSU-MM01 and RegDB datasets. The Rank-1 and mAP obtained from each group of experiments are shown in Table 6. It can be seen that using the lightweight modality generator, incorporating Convolutional Block Attention Module, and introducing Gradient Centralization simultaneously, the performance of the network model on the SYSU-MM01 and RegDB datasets is the best, with Rank-1 improving by 7.12% and 6.34%, respectively; MAP increased by 4.00% and 6.05%, respectively. The performance disparity of the network model on the SYSU-MM01 and RegDB datasets might be attributed to the variance in the sample size of the two datasets.

### 4.5. Visualization Analysis

**Figure 6:**
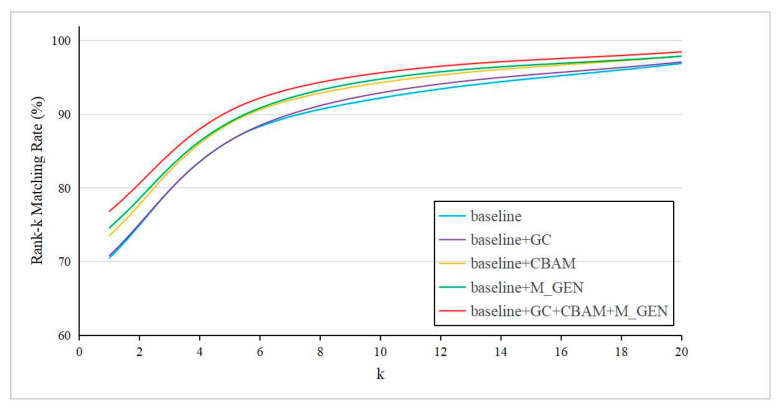
CMC curves of five methods on RegDB dataset.

The CMC of the proposed method on the RegDB dataset is shown in Figure 6. It can be seen that after using the lightweight modality generator, incorporating the Convolutional Block Attention Module, and introducing Gradient Centralization, the network model can perform better on the dataset. This indicates that the proposed method can effectively reduce the modality differences between visible images and infrared images, extract more representative and generalized pedestrian features, and improve the accuracy of person re-identification.

**Figure 7:**
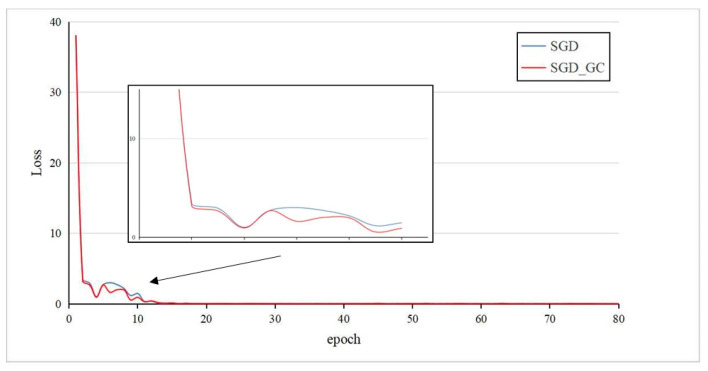
Loss curves before and after introducing Gradient Centralization on RegDB dataset.

The loss curve of the proposed method on the RegDB dataset is shown in Figure 7. It can be seen that after introducing the Gradient Centralization algorithm into the Stochastic Gradient Descent optimizer, the loss of the first epoch is significantly reduced, and the convergence speed of the network model is accelerated. This indicates that the proposed method can improve the convergence speed and generalization ability of the network model.

**Figure 8:**
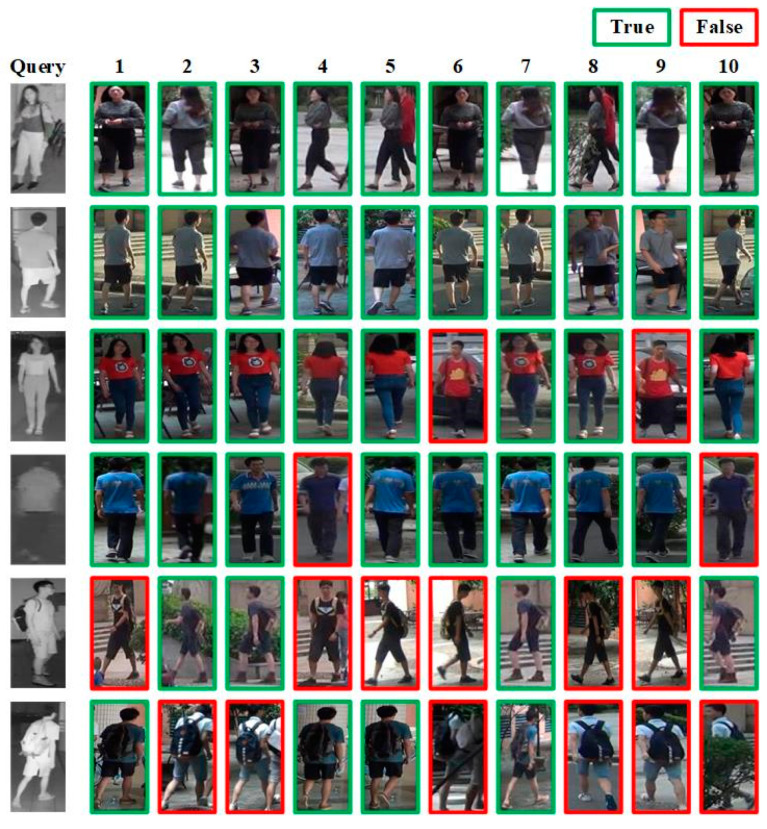
Visualization results output on the SYSU-MM01 dataset.

**Figure 9:**
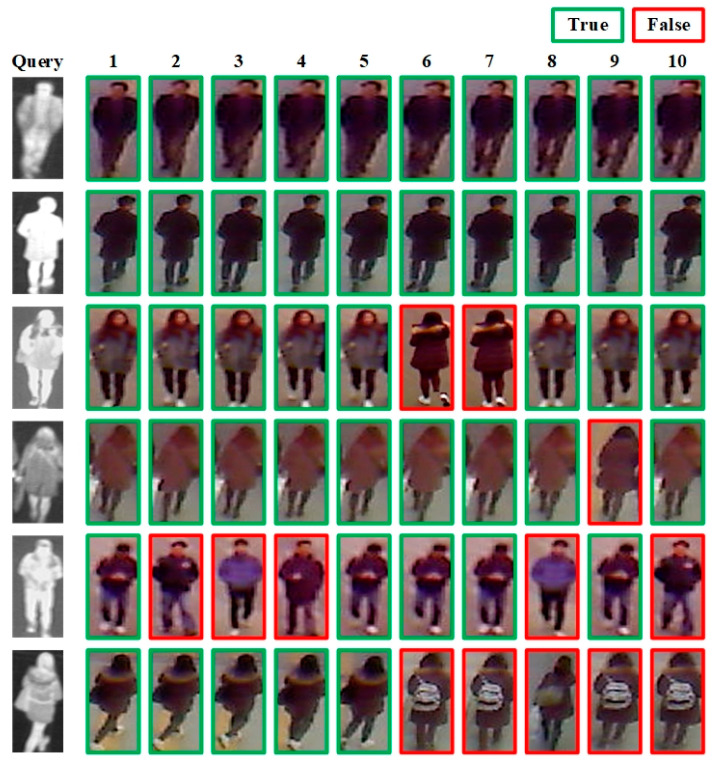
Visualization results output on the RegDB dataset.

The visualization results of the proposed method on the SYSU-MM01 and RegDB datasets are shown in Figure 8 and Figure 9, respectively. Among them, query is the image we want to search, and images 1–10 are the query results returned by the network model from the image database. The images with green borders are considered positive samples, meaning that they belong to the same pedestrian entity as the query image. In contrast, the images with red borders are considered negative samples, meaning that they do not belong to the same pedestrian entity as the query image.

It can be seen that for the simple cases of querying normal front or back infrared pedestrian images, the proposed method can basically retrieve all the correct samples. For more challenging cases, such as querying poor-quality infrared pedestrian images or infrared pedestrian images with occlusions such as backpacks, the proposed method may confuse some samples that are difficult to distinguish. This might be attributed to the considerable noise present in the queried infrared images and the occlusion of key pedestrian features such as backpacks, which leads to the inability of the network model to acquire comprehensive pedestrian-feature information.

## 5. Conclusions

In this paper, we proposed a novel cross-modality person re-identification method that integrates modality generation and feature enhancement. Firstly, the lightweight modality generator is used to generate intermediate modalities between visible images and infrared images, enabling the network model to fully extract shared features between visible images and infrared images; then, the Convolutional Block Attention Module (CBAM) is incorporated into the backbone network ResNet50, and key feature information is weighted and enhanced sequentially from both channel and spatial dimensions to enhance the efficiency and representation ability of the network model in utilizing pedestrian features; finally, the Gradient Centralization (GC) algorithm is introduced into the Stochastic Gradient Descent (SGD) optimizer to improve the convergence speed and generalization ability of the network model. We tested and evaluated the network model on the SYSU-MM01 and RegDB datasets, and the Rank-1 reached 54.62% and 77.84%, respectively. The mAP reached 51.65% and 86.18%, respectively. The above results demonstrate the superiority of the proposed cross-modality person re-identification method.

From the results of this article, it can be observed that it is worth investigating how to improve the computational efficiency of the network model through optimizing the structure and parameters. Additionally, how to employ large language models to facilitate the development of cross-modality person re-identification is also a challenging task. In future research, on the one hand, we will explore efficient cross-modality person re-identification under limited computing resources through techniques such as model compression and pruning. On the other hand, we will explore approaches to enhance the overall performance and robustness of the network model by utilizing the rich contextual information and prior knowledge provided by large language models.

## Figures and Tables

**Table 1:** Dataset condition.

Datasets	Number of Pedestrians	Number of Visible Cameras	Number of Infrared Cameras	Number of Visible Images	Number of Infrared Images
SYSU-MM01	491	4	2	30,071	15,792
RegDB	412	1	1	4120	4120

**Table 2:** Performance comparison of different methods on SYSU-MM01 and RegDB datasets.

Method	SYSU-MM01		RegDB	
	Rank-1	mAP	Rank-1	mAP
Zero-Padding [6]	14.80	15.95	17.75	18.90
HCML [30]	14.32	16.16	24.44	20.08
BDTR [31]	27.82	28.42	34.62	33.46
HSME [32]	20.68	23.12	50.85	47.00
D^2^RL [12]	28.90	29.20	43.40	44.10
MAC [33]	33.26	36.22	36.43	37.03
MSR [34]	37.35	38.11	48.43	48.67
AlignGAN [13]	42.40	40.70	57.90	53.60
Hi-CMD [35]	34.90	35.90	70.93	66.04
XIV-ReID [18]	49.92	50.73	62.21	60.18
baseline (AGW)	47.50	47.65	70.50	80.13
baseline + M_GEN + CBAM + GC (ours) ^1^	54.62	51.65	76.84	86.18

^1^ In this table, M_GEN is an abbreviation for the modality generator, CBAM is an abbreviation for Convolutional Block Attention Module, and GC is an abbreviation for Gradient Centralization, as is the case in subsequent tables.

**Table 3:** Performance comparison of models using lightweight modality generator on SYSU-MM01 and RegDB datasets.

Method	SYSU-MM01		RegDB	
	Rank-1	mAP	Rank-1	mAP
baseline	47.50	47.65	70.50	80.13
baseline + M_GEN	52.97	48.81	74.59	82.52

**Table 4:** Performance comparison of models incorporating Convolutional Block Attention Module on SYSU-MM01 and RegDB datasets.

Method	SYSU-MM01		RegDB	
	Rank-1	mAP	Rank-1	mAP
baseline	47.50	47.65	70.50	80.13
baseline + CBAM	51.39	48.47	73.51	82.23

**Table 5:** Performance comparison of models introducing Gradient Centralization on SYSU-MM01 and RegDB datasets.

Method	SYSU-MM01		RegDB	
	Rank-1	mAP	Rank-1	mAP
baseline	47.50	47.65	70.50	80.13
baseline + GC	47.59	47.88	70.81	80.50

**Table 6:** Results of ablation experiments on SYSU-MM01 and RegDB datasets.

Method	SYSU-MM01		RegDB	
	Rank-1	mAP	Rank-1	mAP
baseline	47.50	47.65	70.50	80.13
baseline + GC	47.59	47.88	70.81	80.50
baseline + CBAM	51.39	48.47	73.51	82.23
baseline + M_GEN	52.97	48.81	74.59	82.52
baseline + GC + CBAM	51.58	49.27	73.24	81.92
baseline + GC + M_GEN	52.91	50.04	75.41	84.54
baseline + CBAM + M_GEN	53.86	50.63	75.76	85.89
baseline + GC + CBAM + M_GEN	54.62	51.65	76.84	86.18

## Data Availability

The data that support the findings of this study are available online. These datasets were derived from the following public resources: [SYSU-MM01, RegDB].
